# Phase-contrast MRI volume flow – a comparison of breath held and navigator based acquisitions

**DOI:** 10.1186/s12880-016-0128-x

**Published:** 2016-03-28

**Authors:** Charlotta Andersson, Johan Kihlberg, Tino Ebbers, Lena Lindström, Carl-Johan Carlhäll, Jan E. Engvall

**Affiliations:** Center for Medical Image Science and Visualization, Linkoping University, SE-581 83 Linkoping, Sweden; Department of Clinical Physiology, Linkoping University, SE-603 79 Norrkoping, Sweden; Department of Diagnostic Radiology, Linkoping University, SE-581 85 Linkoping, Sweden; Department of Medical and Health Sciences, Linkoping University, SE-581 83 Linkoping, Sweden; Department of Clinical Physiology, Linkoping University, SE-581 85 Linkoping, Sweden

**Keywords:** Phase-contrast flow, Magnetic resonance imaging, Segmentation, 2D Doppler echocardiography

## Abstract

**Background:**

Magnetic Resonance Imaging (MRI) 2D phase-contrast flow measurement has been regarded as the gold standard in blood flow measurements and can be performed with free breathing or breath held techniques. We hypothesized that the accuracy of flow measurements obtained with segmented phase-contrast during breath holding, and in particular higher number of k-space segments, would be non-inferior compared to navigator phase-contrast. Volumes obtained from anatomic segmentation of cine MRI and Doppler echocardiography were used for additional reference.

**Methods:**

Forty patients, five women and 35 men, mean age 65 years (range 53–80), were randomly selected and consented to the study. All underwent EKG-gated cardiac MRI including breath hold cine, navigator based free-breathing phase-contrast MRI and breath hold phase-contrast MRI using k-space segmentation factors 3 and 5, as well as transthoracic echocardiography within 2 days.

**Results:**

In navigator based free-breathing phase-contrast flow, mean stroke volume and cardiac output were 79.7 ± 17.1 ml and 5071 ± 1192 ml/min, respectively. The duration of the acquisition was 50 ± 6 s. With k-space segmentation factor 3, the corresponding values were 77.7 ml ± 17.5 ml and 4979 ± 1211 ml/min (*p* = 0.15 vs navigator). The duration of the breath hold was 17 ± 2 s. K-space segmentation factor 5 gave mean stroke volume 77.9 ± 16.4 ml, cardiac output 5142 ± 1197 ml/min (*p* = 0.33 vs navigator), and breath hold time 11 ± 1 s. Anatomical segmentation of cine gave mean stroke volume and cardiac output 91.2 ± 20.8 ml and 5963 ± 1452 ml/min, respectively. Echocardiography was reliable in 20 of the 40 patients. The mean diameter of the left ventricular outflow tract was 20.7 ± 1.5 mm, stroke volume 78.3 ml ± 15.2 ml and cardiac output 5164 ± 1249 ml/min.

**Conclusions:**

In forty consecutive patients with coronary heart disease, breath holding and segmented k-space sampling techniques for phase-contrast flow produced stroke volumes and cardiac outputs similar to those obtained with free-breathing navigator based phase-contrast MRI, using less time. The values obtained agreed fairly well with Doppler echocardiography while there was a larger difference when compared with anatomical volume determinations using SSFP (steady state free precession) cine MRI.

## Background

The generation of cardiovascular flow has been said to be the essence of cardiology [1]. Unfortunately, in clinical practice, the applicability of methods used to determine flow may be restricted by the pre-existing condition of the patient. Various techniques have been favoured and later abandoned such as indicator dilution with indocyanine green, while others, e.g. thermodilution, have withstood the test of time. Completely non-invasive determination of stroke volume with echocardiography and Doppler recording is versatile and readily available at the bedside, but the calculation rests on a number of assumptions such as a circular geometry of the left ventricular outflow tract [2] and a spatially flat flow profile [3]. MRI phase-contrast flow measurement has been regarded as the gold standard since it can address issues of temporally as well as spatially varying flows [4, 5]. However, MRI velocity measurements are sensitive to magnetic field inhomogeneities, concomitant gradient effects, and eddy current effects that are only partly compensated for [6]. Gatehouse suggested that an error of 5 % could be acceptable in clinical practice, which would be equivalent to 4 ml when the stroke volume is 80 ml and 250 ml when cardiac output is 5000 ml/min [7]. Previous work has suggested that the size of the great vessels is the most important factor that determines baseline phase offset [8]. Furthermore, MRI collects flow data from several heart beats and cannot measure beat-by-beat variation, except when using techniques of reduced sampling such as the pencil beam technique for real-time flow velocity [9]. In busy daily practice, sampling is performed during a short breath hold that may introduce some errors due to physiological effects on cardiac filling and effects of averaging when using segmented k-space sampling methods [10–12]. The extent of these effects is influenced by the length of the breath holding, which in its turn depends on heart rate and scanner settings. To avoid the physiological effects of holding breath, the obvious alternative would be sampling during free breathing [13]. However, due to a longer sampling time, this will add a component of temporal averaging.

Patients are at times dyspnoeic and are frequently limited in their capacity to hold their breath which would favour the use of the free breathing technique or an alternative with the shortest breath hold. Since there is no agreement on which MRI phase-contrast technique to prefer, we hypothesized that the accuracy of volume flow measurements obtained with segmented phase-contrast during breath holding, and in particular higher number of k-space segments, would be non-inferior compared to navigator phase-contrast which has potential to become a standard of reference. Volumes obtained from anatomic segmentation of cine MRI and Doppler echocardiography were used for additional reference.

## Methods

Forty patients, five women and 35 men, mean age 65 years (range 53–80), were randomly selected and gave written consent to the study (Table 1), which was approved by the Regional Ethics Committee in Linkoping, Dnr M216-09. All were part of the Doppler-cip study and had undergone a cardiac MRI scan and a transthoracic Doppler echocardiography within 2 days [14, 15]. MRI was performed with a Philips Achieva Nova Dual R 3.2, 1.5 T system, with a 5-element phased array cardiac coil (Philips Healthcare, Best, the Netherlands) and Doppler echocardiography with a GE Vivid 7 ultrasound scanner (GE Healthcare, Horten, Norway).

**Table 1:** Demographic data for the patients in the study

Demographic data	Age, mean (SD), years	65 (7)
	Female, n (%)	6 (15)
	Body mass index, mean (SD) kg/m2	26.5 (3.7)
Medical history	Diabetes, n (%)	8 (20)
	Hypertension, n (%)	14 (35)
	Myocardial infarction, n (%)	22 (55)
	CABG, n (%)	9 (23)
	PCI, n (%)	18 (45)
	Moderate MR (1) or AR (1) at Doppler	6 (15)

The MR flow slice was positioned transverse to the ascending aorta cranial to the sino-tubular junction where the flow is parallel to the long-axis of the body in order to obtain through-plane flow perpendicular to the slice. The acquisition was retrospectively gated to the EKG using the following parameters: slice thickness 8 mm, field of view (FOV) 320 × 260 mm, acquisition matrix 128 × 104 (reconstructed to 256 × 256), sensitivity encoding (SENSE) factor 2, velocity encoding 200 cm/s, repetition time 4.6 ms and echo time 2.7 ms. The effect on scan duration of using three different k-space segmentation factors (TFE) was studied. Scans with TFE factor of 5 and 3 were acquired in breath hold while free breathing with navigator triggering (6 mm gate and track window, continuous level drift) was used for TFE 1. The number of reconstructed cardiac phases was adjusted to the heart rate and k-space segmentation factor, e.g. from 16 (at TFE 5 and 80 beats/min) to 80 (at TFE 1 and 40 beats/min). Depending on the heart rate, the duration of breath holding could vary between 9 s (TFE 5, 80 beats/min) up to 28 s (TFE 3, 40 beats/min). The navigator scan took from 42 s (TFE 1, 80 beats/min) to 1:24 min (TFE 1, 40 beats/min). All velocity data was corrected for concomitant gradient effects on the scanner as suggested by Bernstein et al [16]. Background offset due to eddy current effects was corrected on the scanner by using the default local phase correction algorithm, which is based on an optimized spatial low pass filter (Philips Healthcare internal white paper April 12, 2012).

In addition to flow, anatomical volume measurements were performed on cine SSFP short axis images covering the left ventricle from base to apex. Slice thickness was 8 mm and slice gap 2 mm. Temporal resolution ranged between 26 and 41 ms (30 acquired phases).

Data analysis was performed on a separate workstation using software from the vendor (Philips Extended MR Workspace, version 2.6.6.3). For flow measurements, an elliptical template covering the aortic perimeter was applied and adapted to the vessel using an active contour-seeking algorithm. After manual correction, the segmentation was migrated to adjacent time frames using the active contour-seeking algorithm until the entire cardiac cycle was covered. Manual corrections were applied whenever necessary. The volume flow was calculated by temporal integration of the velocities within the segmented area, using the antegrade flow component (all forward flow components in the entire heart cycle, without deducting backward flow components) to facilitate a comparison with Doppler echocardiography and cine MRI. Cardiac output was computed as stroke volume multiplied with heart rate. Differences in heart rate between the three flow acquisitions were calculated and the largest individual difference averaged between all patients.

Anatomical MRI-based stroke volume was determined by manually segmenting the stack of short axis images of the left ventricle, in end diastole as well as in end systole. End systole was determined from the smallest ventricular area of a mid-ventricular slice, or, in case of dyssynchrony, from the time point closest to end systole determined from aortic closure in the apical long axis view [17]. The papillary muscles were included in the volume of the left ventricular cavity and the outflow tract was excluded [17]. The measurements were done in duplicate and the mean value was used in the comparisons. The duplicate measurements were used to report intraobserver reproducibility. Interobserver variability was reported from ten studies segmented by a second observer. Further data on reproducibility have been published elsewhere [14].

All patients underwent Doppler echocardiography. Stroke volume was calculated from the area of the left ventricular outflow tract (LVOT), determined from the inner-edge to inner-edge diameter according to recommendations from the European Association of Cardiovascular Imaging [2], and multiplied with the velocity time integral (VTI) determined at the level of the diameter measurement but not requiring the presence of a valve opening artefact. Pulmonary shadowing preventing the delineation of the LVOT was considered a criterion for excluding the measurement as well as excessive VTI due to placement of the sample volume in the aortic annulus. Since the echocardiogram typically was performed two days after the MRI scan, heart rate differed somewhat which necessitated using cardiac output for the comparisons.

### Statistical analysis

All measurements were reasonably well normally distributed which allowed Student’s *t*-test to be used for tests of statistical significance. A *p*-value of <0.05 was considered significant. For differences between methods, analysis according to Bland-Altman and linear regression was used. Percent values were given based on the difference of the averages. Descriptive statistics were reported as mean values with 1 standard deviation (SD).

## Results

### Stroke volume and cardiac output from phase-contrast MRI

**Fig. 1:**
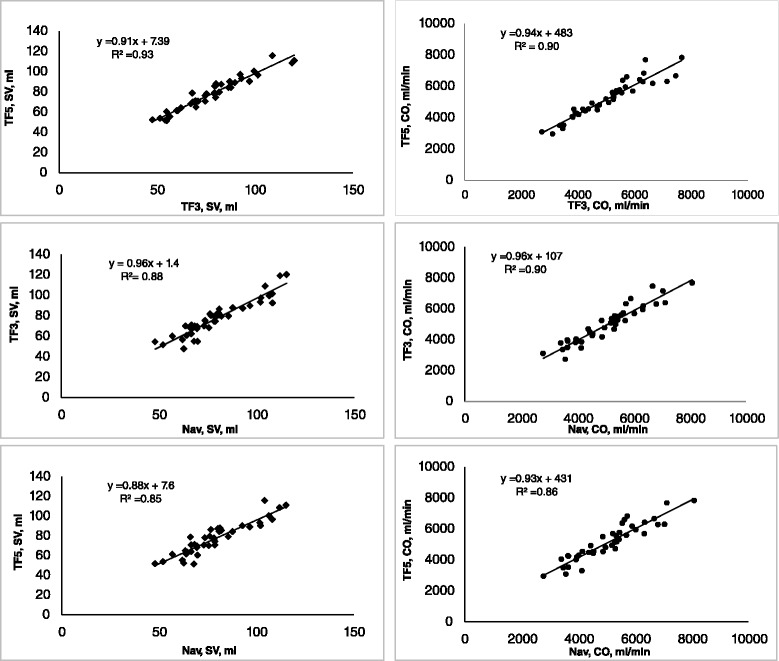
Correlation between the phase-contrast techniques for stroke volume and cardiac output. *N* = 40 for all measurements

**Fig. 2:**
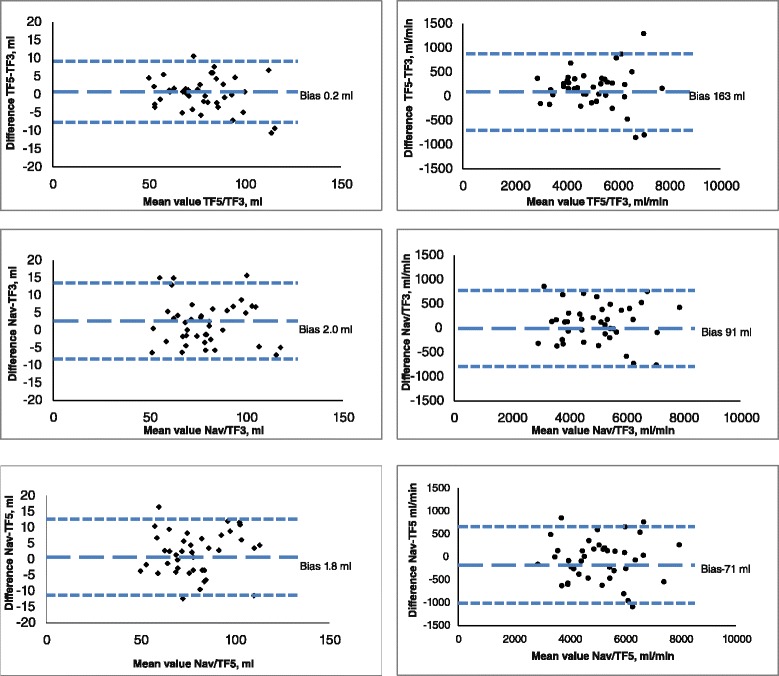
Bland-Altman diagram depicting stroke volume and cardiac output for the three phase-contrast techniques. *N* = 40 for all measurements. Bias and limits of agreements are given in dashed blue

In navigator based, EKG-gated free-breathing phase-contrast flow, mean stroke volume and cardiac output were 79.7 ± 17.1 ml and 5071 ± 1192 ml/min, respectively (Table 2). The duration of the acquisition was 50 ± 6 s. With TFE 3, the corresponding values were 77.7 ml ± 17.5 ml and 4979 ± 1211 ml/min. The duration of the breath hold was 17 ± 2 s. Using TFE 5, mean stroke volume, cardiac output and breath hold time was 77.9 ± 16.4 ml, 5142 ± 1197 ml/min, and 11 ± 1 s, Table 2. The mean difference between the three methods is depicted in Table 3 and Figs. 1 and 2 (regression and Bland-Altman). Flow with k-space segmentation factor 5 did not differ from k-space segmentation factor 3 (*p* = 0.76) for stroke volume, but was larger for cardiac output (*p* = 0.013). Navigator based flow did not differ from TF3 or TF5 for cardiac output, but was barely larger for navigator vs TF3 for stroke volume (*p* = 0.046).

**Table 2:** Stroke volume and cardiac output results

	Stroke volume (ml)	Scan duration (s)	Heart rate (beats/min)	Cardiac output (ml/min)
Cine segmented	91.2 +/− 20.8		66 +/− 8.8	5964 +/− 1452
Phase contrast TF5	77.9 +/− 16.4	11 +/− 1	66 +/− 9.4	5142 +/− 1197
Phase contrast TFS	77.7 +/− 17.5	17 +/− 2	64 +/− 8.6	4979 +/− 1211
Phase contrast (navigator)	79.7 +/− 17.1	50 +/− 6	64 +/− 7.8	5071 +/− 1192

Mean value and SD (standard deviation) for stroke volume and cardiac output for anatomical calculation from cine MRI and the three phase-contrast techniques

**Table 3:** Stroke volume and cardiac output, mean difference between the three phase-contrast techniques

Comparison of methods	Stroke volume (ml)	Cardiac output (ml/min)
	Mean difference, +/−STD	Mean difference, +/− STD
TF5/TF3	0.2	163
	+/− 4.6	+/− 395
	*p* = 0.76	*p* = 0.013
Nav/TF3	2.0	92
	+/− 6.1	+/− 394
	*p* = 0.046	*p* = 0.15
Nav/TF5	1.8	−71
	+/− 6.8	+/− 462
	*p* = 0.10	*p* = 0.33

The mean difference between the phase-contrast based methods. The difference between TF5 (k-space segmentation factor 5) and TF3 (k-space segmentation factor 3) was non-significant (*p* = 0.76) for stroke volume but significant for cardiac output (*p* = 0.013). The difference between navigator vs TF3 as well as navigator vs TF5 was non-significant for cardiac output but barely significant for navigator vs TF3 for stroke volume

### Stroke volume and cardiac output based on left ventricular volumes from cine SSFP MRI

Mean stroke volume and cardiac output were 91.2 ± 20.8 ml and 5963 ± 1452 ml/min, respectively, Table 1. Intraobserver reproducibility expressed as coefficient of variation (SD divided by the mean) was 4 % for LVEDV, 8 % for LVESV and 7 % for stroke volume. The corresponding values for interobserver variability calculated from segmenting 10 patients was 6.4 % for LVEDV, 11.2 % for LVESV and 7.6 % for stroke volume. Interobserver bias and limits of agreement for stroke volume was in this subsample 1.9 ± 13.4 ml.

### Doppler Echocardiography for flow measurement

**Fig. 3:**
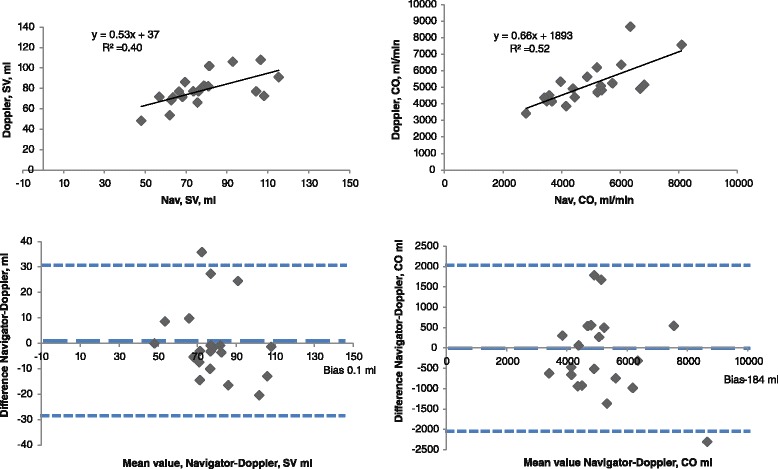
Correlation and Bland-Altman diagram for Doppler-echocardiography and navigator-based phase-contrast MRI for stroke volume and cardiac output. *N* = 20. Bias and limits of agreements are given in dashed blue

Twenty patients were excluded due to either unreliable diameter measurements of the left ventricular outflow tract or inappropriate placement of the sample volume causing an overestimation of the velocity time integral. In the remaining 20 patients, the mean diameter of the left ventricular outflow tract was 20.7 ± 1.5 mm, stroke volume 78.3 ml ± 15.2 ml and cardiac output 5164 ± 1249 ml/min. A comparison with navigator flow data is given in Fig. 3.

### Effects of heart rate

Even over shorter periods of time, heart rate varies with anxiety/arousal of the patient. In this study, individual heart rates differed substantially between the different acquisitions, the largest difference in a particular patient being 31 beats/min. The lowest difference between any of the acquisitions in one individual was 4 beats/min. The average largest difference for all individuals was 11.3 ± 5.9 beats/min, but in a group-wise comparison, these individual differences are not apparent on the mean values (Table 2).

## Discussion

This study shows that measuring aortic flow during short breath holds is feasible, despite concern that breath holding might affect cardiac filling. All measurement methods are prone to biological variation and inherent variation in accuracy and repeatability [18]. Some measurements require the full cooperation of patients, at times exceeding their limits e.g. when holding breath in obstructive pulmonary disease. For a long time, MRI phase-contrast has been considered the gold standard in the non-invasive determination of stroke volume and cardiac output [19, 20]. However, MRI can be executed in many different ways. In general, methods that shorten the time required for the collection of data are attractive since they ease the demands on the patient thereby facilitating work flow.

In this study comparing free breathing and breath hold recording of aortic flow, we found that the mean difference was below 2 ml (2.5 %) for stroke volume and below 163 ml/min (3.2 %) for cardiac output, which we consider acceptable for everyday clinical practice (Fig. 2, Table 3). Individual differences are also within clinically acceptable levels, with 55–75 % in the interval ±5 ml for stroke volume and 67–82 % ±500 ml/min for cardiac output in Bland-Altman analysis (Fig. 2). Close to one minute acquisition time for the free breathing sequence may seem short, but the breath held techniques are considerably quicker, without significant errors in measurement. When choosing between the two levels of k-space segmentation (TFE factors 3 and 5) TFE 5 was 5 s faster which may seem little, but for an ill patient, holding breath for 11 s is a lot easier than holding breath for 16 s.

All patients provided evaluable phase-contrast flow results, with mean values within 2 % to that from the Doppler echocardiography results when available (Table 3). However, in Doppler echocardiography an inability to determine the LVOT diameter and a tendency towards overestimation of VTI caused many exclusions. Three-dimensional techniques have demonstrated that the LVOT is elliptical [2] and the spatial flow profile of the LVOT has been demonstrated to be skewed in healthy individuals and in patients with aortic regurgitation [3]. These conditions may have contributed to an inaccurate determination of stroke volume in the present study.

The largest difference found was between the flow based techniques and the anatomically determined MRI flow volume. Few patients in this study (5 MI, 1 AI) had more than trace mitral or aortic regurgitation (Table 1). It has been hypothesized that the combination of coronary blood flow not being included in the phase-contrast aortic sampling and the presence of unrecognized mitral regurgitation may explain a large part of the difference between phase-contrast and anatomic flow values [17, 21].

### Relation to earlier studies

In healthy volunteers Polte et al found a bias of 12 ml and limits of agreement of 0–24 ml between anatomic and phase-contrast stroke volumes [22]. Likewise, James et al. found 5–7 ml difference in anatomical vs phase-contrast stroke volume that was attributed to coronary flow [23]. Differences between anatomically based measurements were also found in a recent multi-modality study of LVEDV with smaller 2D- and 3D-echo volumes than those obtained with the goldstandard MRI [23, 24]. Even with the high image quality obtained with SSFP and despite use of a meticulous segmentation technique, there will always be need for training [25] to overcome difficulties in the definition of the most basal slice of the left ventricle and the definition of the endocardial border in the presence of trabeculae. Suinesiaputra et al have recently recommended systematic training on a specific dataset to improve on the result of manual segmentation. However, the reproducibility of segmentation in the Doppler-cip study, of which the data here presented is a subset, has been extensively discussed in a previous publication, with interobserver bias and LOA of 8.2 + 7.7 ml for stroke volume [26]. The recommendations on segmentation are still subject to changes [17].

## Conclusions

In forty consecutive patients with coronary heart disease, using breath holding and segmented k-space sampling techniques for phase-contrast flow produced stroke volumes and cardiac outputs similar to those obtained with free breathing navigator based phase-contrast MRI, using less time. The values obtained agreed fairly well with Doppler echocardiography while there was a larger difference when compared with anatomical volume determinations using SSFP MRI.

## Availability of supporting data

Due to statutory provisions regarding data- and privacy protection, the dataset supporting the conclusions of this article is available upon individual request directed to the corresponding author.

## Abbreviations

LVEDV: left ventricular end diastolic volume
MRI: magnetic resonance imaging
SENSE: sensitivity encoding
SSFP: Steady State Free Precession
TFE: turbo field echo
